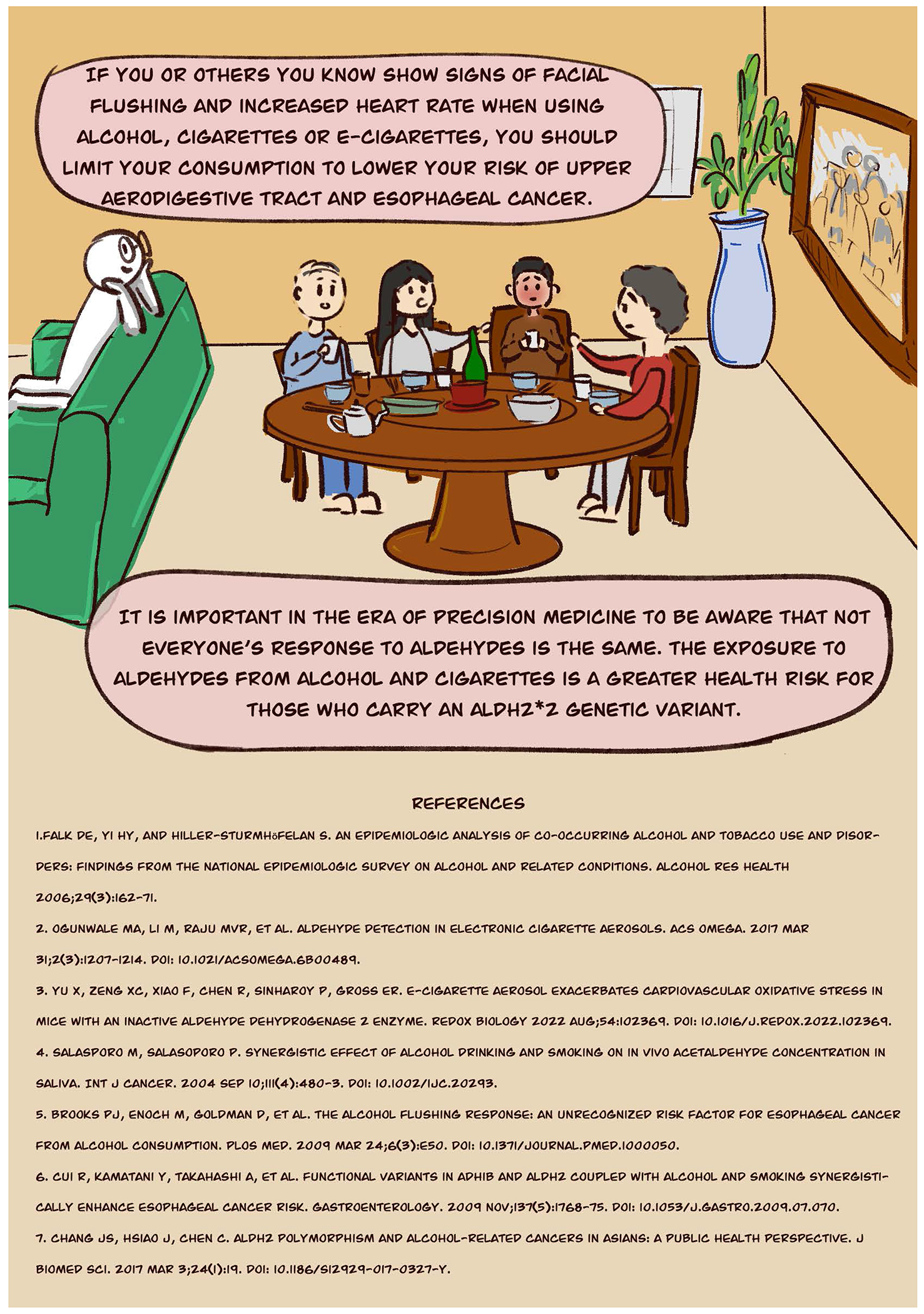# The Alcohol Flush Response

**DOI:** 10.7191/gmr.807

**Published:** 2024-02-22

**Authors:** Isabel Moh, Daniel Simon, Eric R. Gross

**Affiliations:** University of Nevada, Reno, NV USA; Anesthesiology, Perioperative and Pain Medicine, Stanford Medicine, Stanford University, Stanford, CA USA; Anesthesiology, Perioperative and Pain Medicine, Stanford Medicine, Stanford University, Stanford, CA USA

## Abstract

Nearly 540 million people world-wide have facial flushing and an increased heart rate after consuming alcohol. Known as the alcohol flushing response, this reaction to alcohol is a result of a genetic variant in an enzyme aldehyde dehydrogenase 2 (ALDH2), known as ALDH2*2. Mainly carried by those of East Asian descent, the genetic variant is likely the most common genetic variant carried in the world. Carrying this ALDH2*2 genetic variant has important health implications with respect cancer risk which is increased when carriers of the ALDH2*2 genetic variant frequently use of alcohol or tobacco products. This comic explains the alcohol flush response and the health risks associated with alcohol and tobacco use for those who carry an ALDH2*2 variant.

## Background

The alcohol flush response is a reaction to alcohol that causes facial flushing and tachycardia. Mainly prevalent for those of East Asian descent (China, Japan, Korea, and Taiwan), this response to alcohol affects nearly 540 million people worldwide and is caused by an inactive genetic variant in the enzyme aldehyde dehydrogenase 2 (ALDH2) (Gross, 2015).

So why should we care whether people flush after they drink alcohol? This phenotype of flushing after consuming alcohol has important health implications. As described in this comic, the facial flushing that occurs after alcohol consumption is a warning sign that the body cannot break-down a metabolite of alcohol, called acetaldehyde. This alcohol metabolite acetaldehyde in turn accumulates within the body. This accumulation of acetaldehyde is what triggers the physiological effects that produce the phenotype of facial flushing and tachycardia (Chen, 2014).

Acetaldehyde accumulation at the cellular level can trigger DNA damage and modify protein functions by forming aldehyde-induced adducts on DNA and proteins (Heymann, 2018). These adducts that occur with alcohol exposure can lead to changes within a cell that can lead to cancer (Brooks, 2009). Since alcohol is typically a beverage we drink, the alcohol exposure is concentrated within the digestive system – leading to higher risks of cancer particularly of the upper digestive track (mouth and esophagus) due to the limited ability to metabolize the alcohol metabolite acetaldehyde to acetic acid. Importantly, there are other sources of aldehydes besides alcohol. This comic highlights that besides alcohol, another source of aldehyde exposure is cigarettes and e-cigarettes. Aldehydes, including acetaldehyde, formaldehyde, and acrolein are inhaled when using e-cigarettes (Yu, 2022). This exposure to aldehydes that are present in cigarettes or e-cigarettes, when combined with alcohol, can create an additive effect of aldehyde accumulation particularly within the upper digestive tract (Salasporo, 2004).

As aldehydes can be consumed or inhaled, besides aldehydes driving the risk for upper digestive tract cancer, aldehydes also can lead to vascular inflammation (Guo, 2023; Yu, 2022). This inflammation of the vascular system may over time potentially lead to cardiovascular and neurovascular disease (Zhang, 2023).

Due to these health reasons, it is important to relay to the public the risk carried by people that flush after drinking alcohol. We hope that relaying this information through a comic presenting the alcohol flushing response is an additional avenue to reach people about this health risk. Part of our future plans include additional public outreach relaying the health risks associated with the alcohol flushing response.

## Conclusion

The alcohol flushing response is not benign. Frequent exposure to aldehydes, especially when alcohol is combined with aldehyde exposures from sources such as cigarettes or e-cigarettes may increase the risk of developing cancer.

## Figures and Tables

**Figure:**
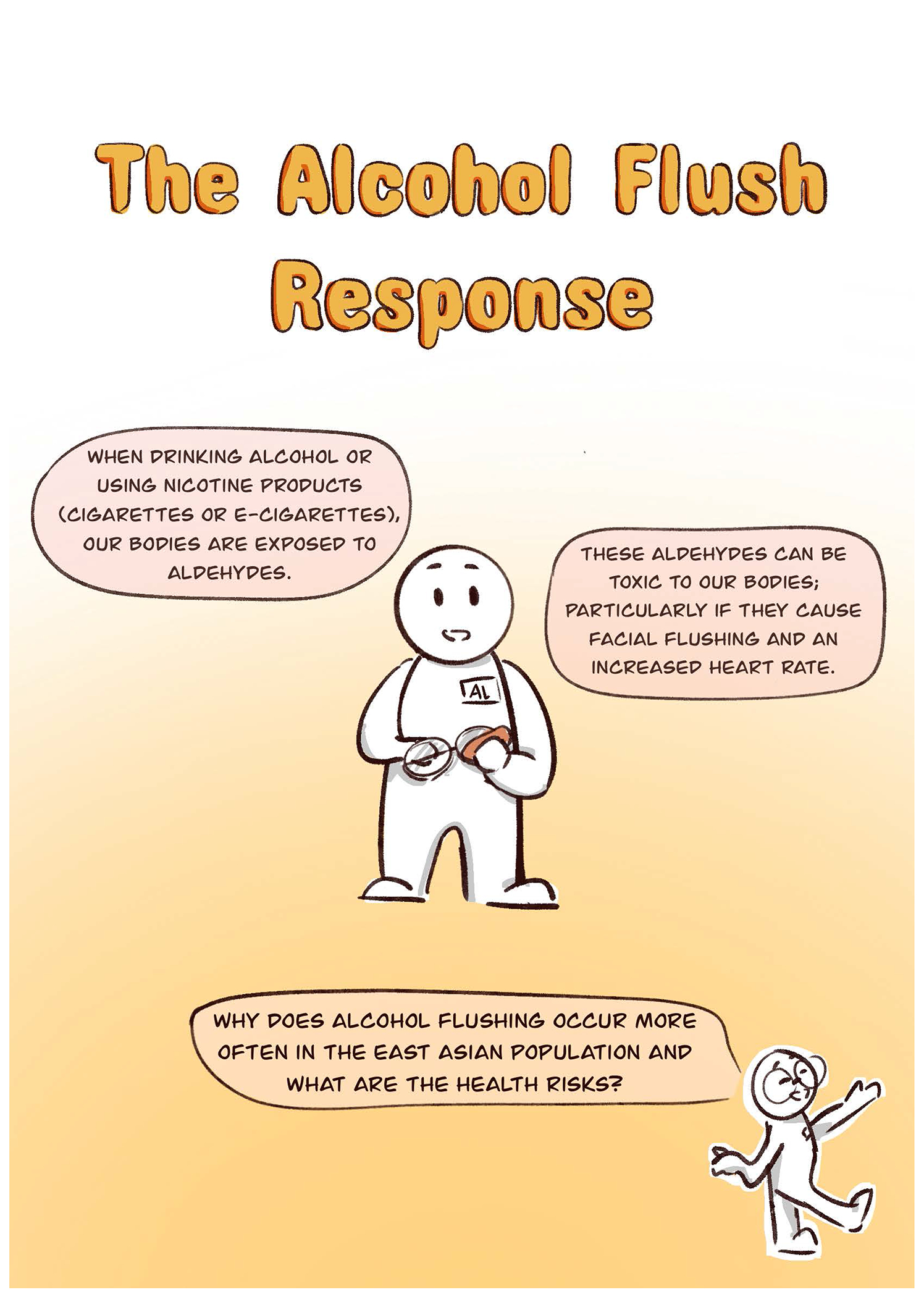


**Figure:**
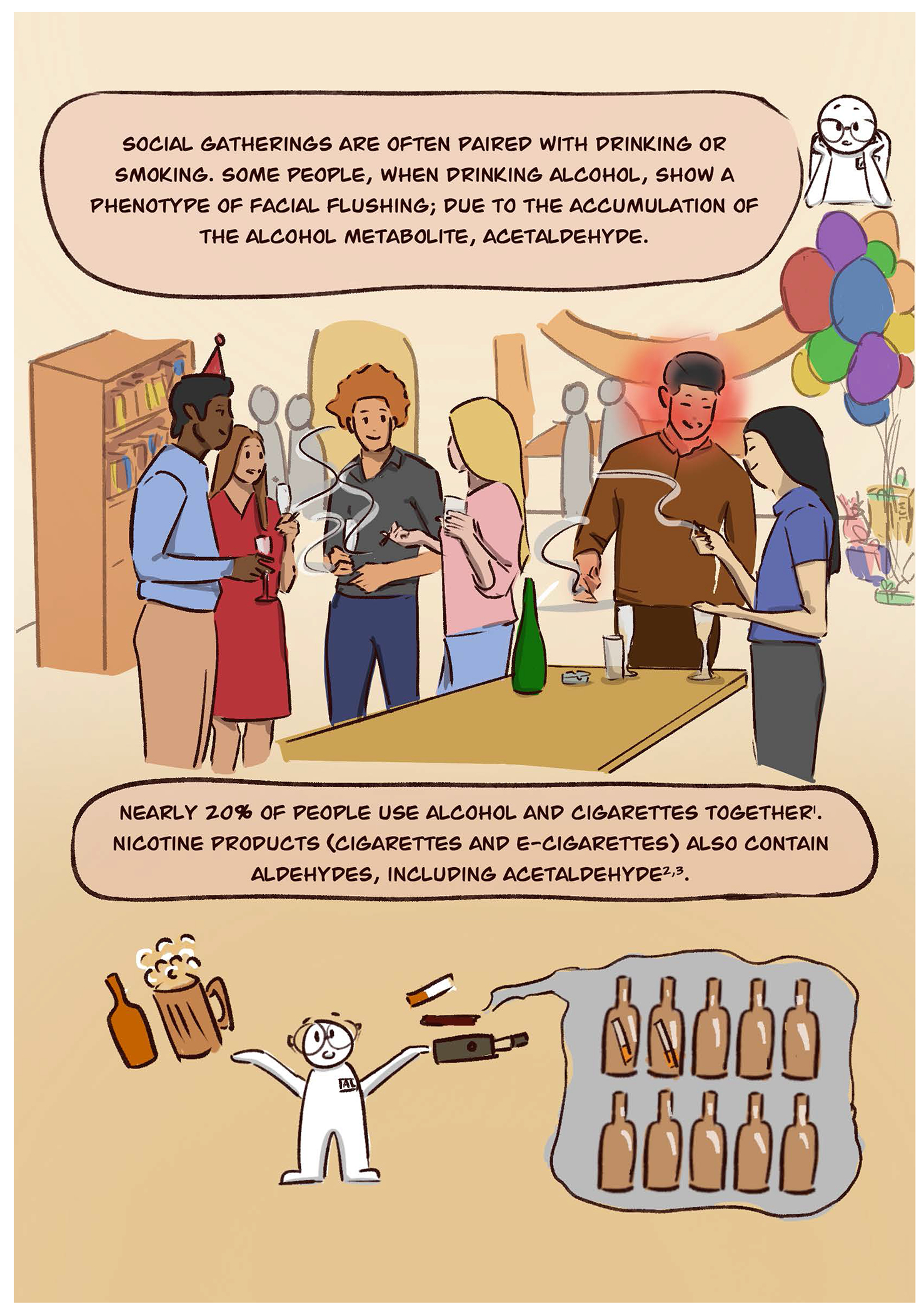


**Figure:**
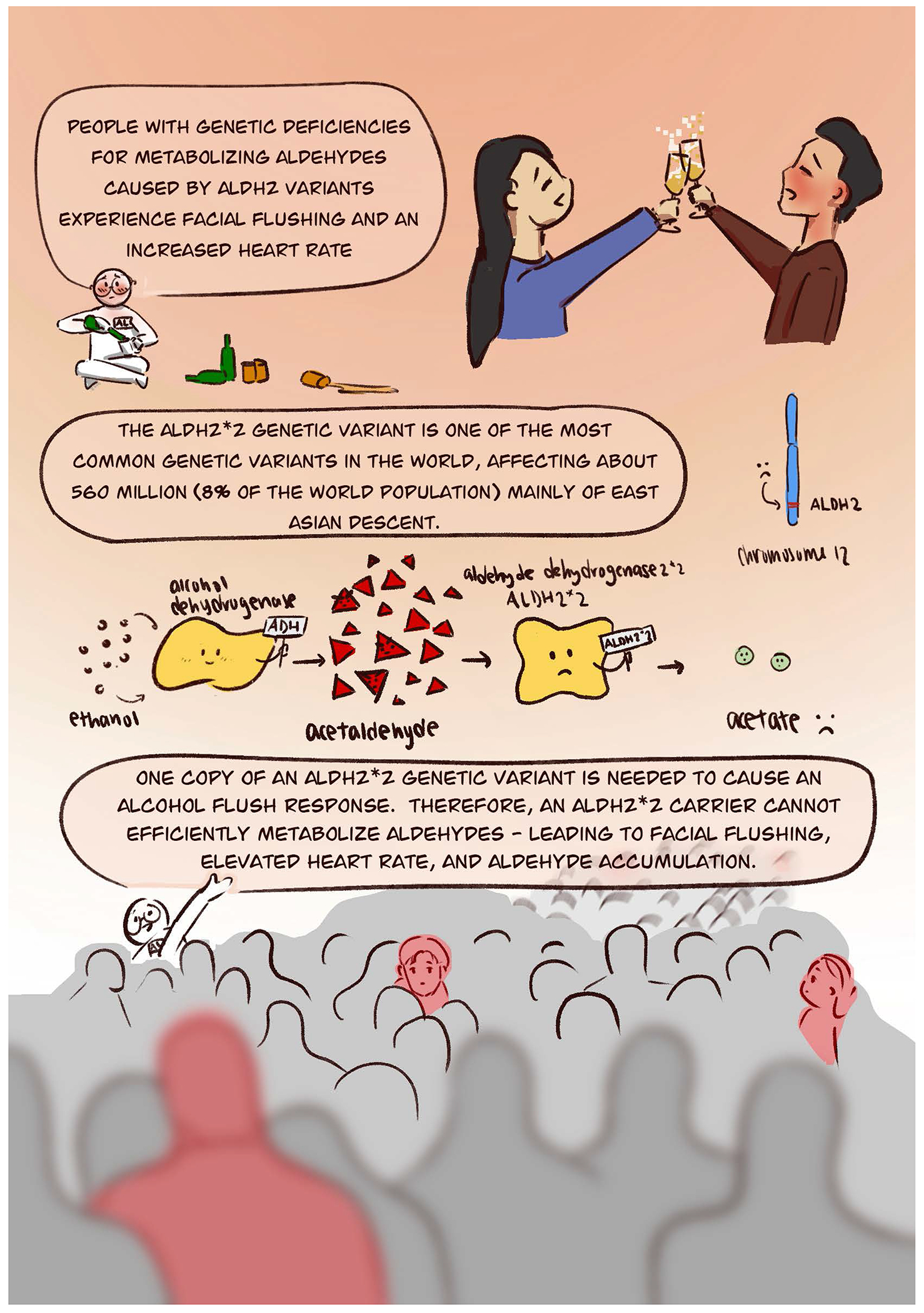


**Figure:**
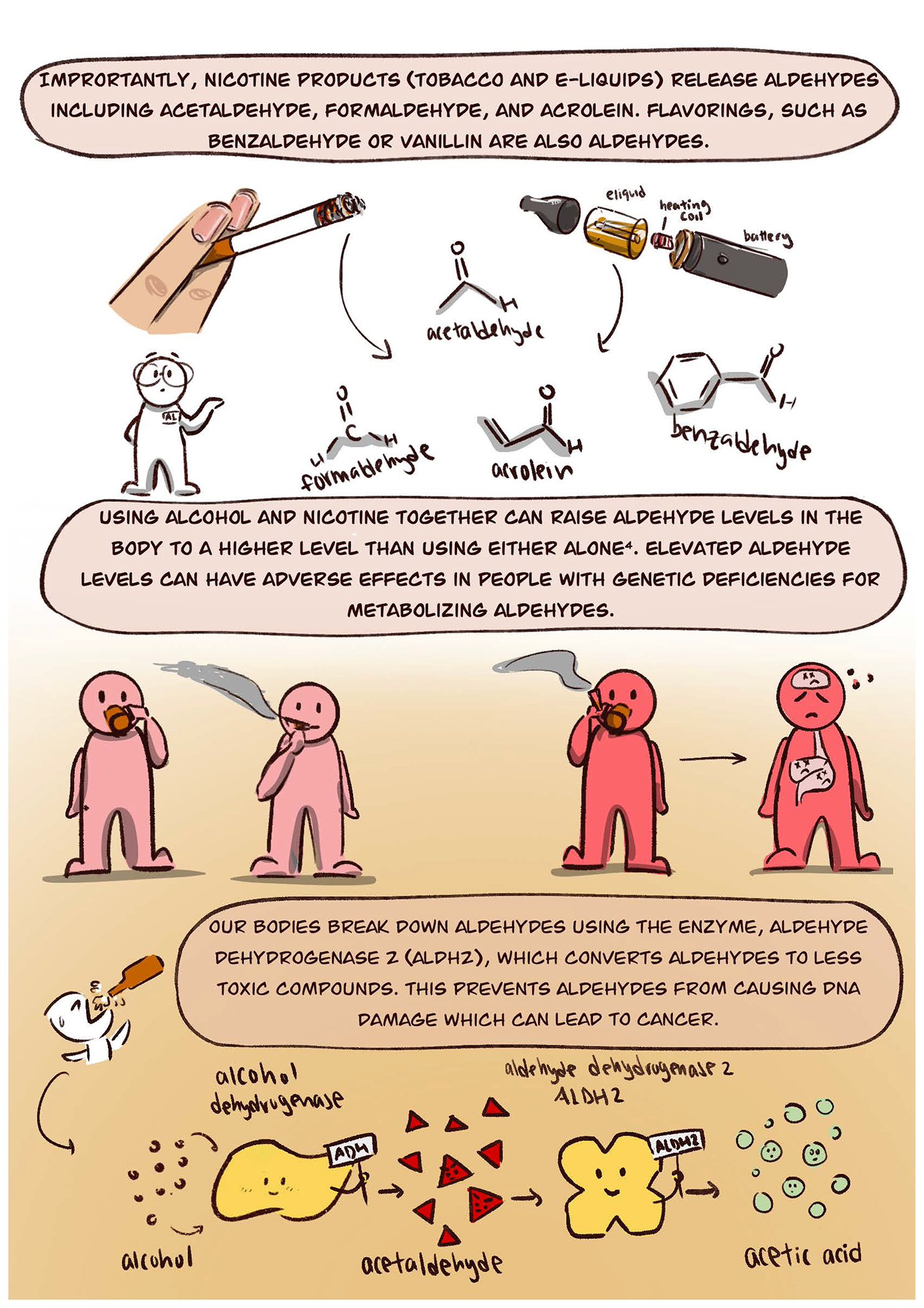


**Figure:**
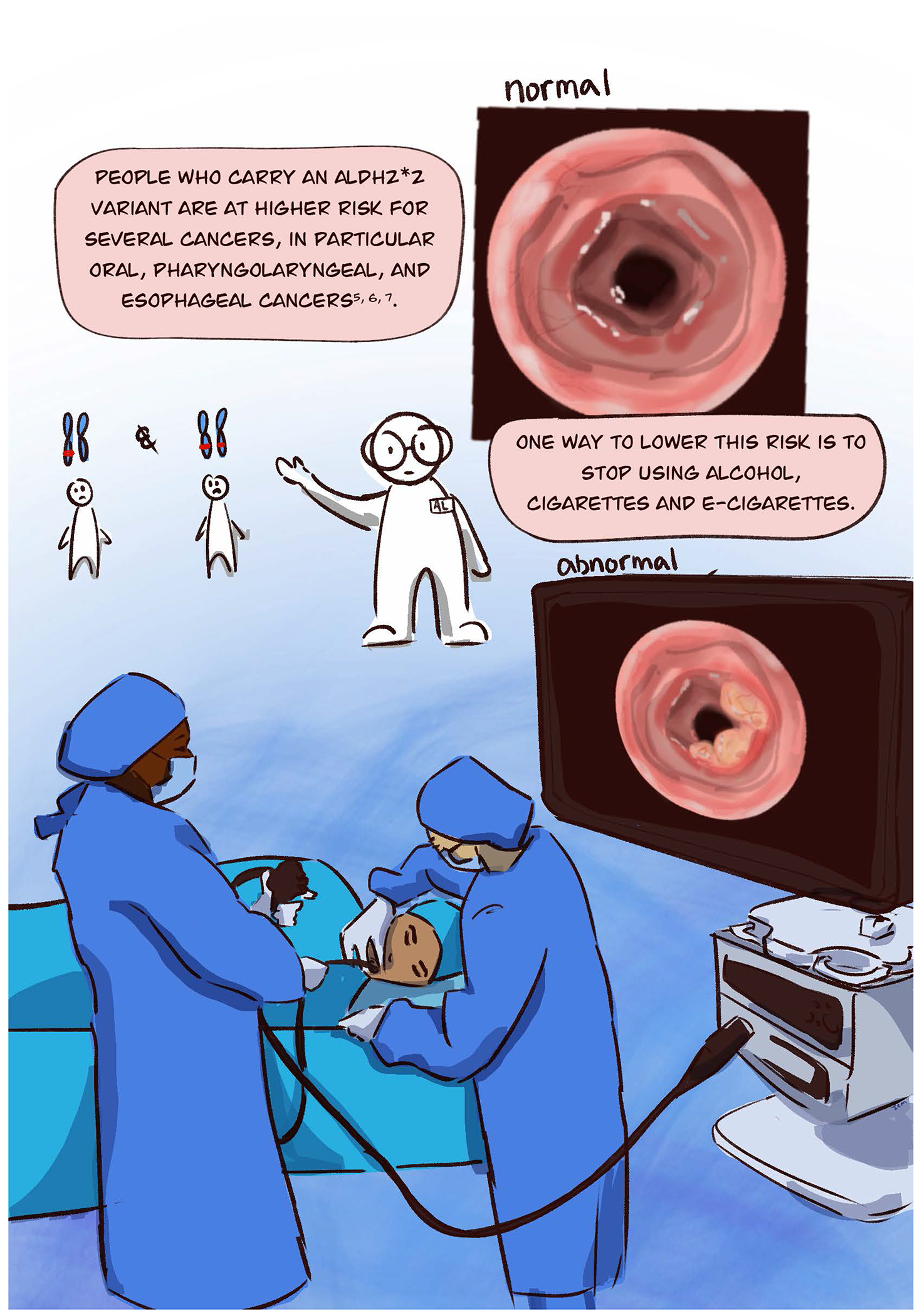


**Figure:**